# Face the Future—Artificial Intelligence in Oral and Maxillofacial Surgery

**DOI:** 10.3390/jcm12216843

**Published:** 2023-10-30

**Authors:** Maximilian F. Miragall, Samuel Knoedler, Martin Kauke-Navarro, Rakan Saadoun, Alex Grabenhorst, Florian D. Grill, Lucas M. Ritschl, Andreas M. Fichter, Ali-Farid Safi, Leonard Knoedler

**Affiliations:** 1 Department of Oral and Maxillofacial Surgery, University Hospital Regensburg, 93053 Regensburg, Germany; 2 Department of Oral and Maxillofacial Surgery, School of Medicine, Technical University of Munich, 81675 Munich, Germany; 3 Division of Plastic Surgery, Department of Surgery, Yale New Haven Hospital, Yale School of Medicine, New Haven, CT 06510, USA; 4 Department of Plastic Surgery, University of Pittsburgh, Pittsburgh, PA 15261, USA; 5 Craniologicum, Center for Cranio-Maxillo-Facial Surgery, 3011 Bern, Switzerland; asafi@outlook.de; 6 Faculty of Medicine, University of Bern, 3010 Bern, Switzerland; 7 Department of Plastic, Hand and Reconstructive Surgery, University Hospital Regensburg, 93053 Regensburg, Germany

**Keywords:** oral and maxillofacial surgery, oral surgery, maxillofacial surgery, OMFS, artificial intelligence, AI, deep learning, machine learning

## Abstract

Artificial intelligence (AI) has emerged as a versatile health-technology tool revolutionizing medical services through the implementation of predictive, preventative, individualized, and participatory approaches. AI encompasses different computational concepts such as machine learning, deep learning techniques, and neural networks. AI also presents a broad platform for improving preoperative planning, intraoperative workflow, and postoperative patient outcomes in the field of oral and maxillofacial surgery (OMFS). The purpose of this review is to present a comprehensive summary of the existing scientific knowledge. The authors thoroughly reviewed English-language PubMed/MEDLINE and Embase papers from their establishment to 1 December 2022. The search terms were (1) “OMFS” OR “oral and maxillofacial” OR “oral and maxillofacial surgery” OR “oral surgery” AND (2) “AI” OR “artificial intelligence”. The search format was tailored to each database’s syntax. To find pertinent material, each retrieved article and systematic review’s reference list was thoroughly examined. According to the literature, AI is already being used in certain areas of OMFS, such as radiographic image quality improvement, diagnosis of cysts and tumors, and localization of cephalometric landmarks. Through additional research, it may be possible to provide practitioners in numerous disciplines with additional assistance to enhance preoperative planning, intraoperative screening, and postoperative monitoring. Overall, AI carries promising potential to advance the field of OMFS and generate novel solution possibilities for persisting clinical challenges. Herein, this review provides a comprehensive summary of AI in OMFS and sheds light on future research efforts. Further, the advanced analysis of complex medical imaging data can support surgeons in preoperative assessments, virtual surgical simulations, and individualized treatment strategies. AI also assists surgeons during intraoperative decision-making by offering immediate feedback and guidance to enhance surgical accuracy and reduce complication rates, for instance by predicting the risk of bleeding.

## 1. Introduction

Artificial intelligence (AI) is a rapidly advancing discipline that harnesses the power of computer science and vast datasets to enhance problem-solving capabilities. By mimicking human intelligence, AI systems offer a plethora of benefits to individuals in their daily lives. From eliminating spam emails to cutting-edge smartwatches differentiating between routine and aerobic activities through accelerometer sensors and personalized product recommendations by online retailers, AI has seamlessly integrated into societies worldwide [1,2].

Moreover, AI has made various contributions to the healthcare industry, revolutionizing the way medical services are delivered. The 4P model of medicine–Predictive, Preventive, Personalized, and Participatory–has become increasingly obtainable due to the incorporation of intelligent medical technologies [3]. Moreover, AI-powered applications play a crucial role in promoting optimal therapeutic compliance, ensuring patients adhere to prescribed treatment plans and achieve better health outcomes [4,5,6]. These advances can also be applied to specific specialties within the field of medicine.

Within the field of healthcare, oral and maxillofacial surgery (OMFS) stands to benefit significantly from the integration of AI. This specialized discipline of medicine deals with surgical procedures related to the mouth, jaws, face, and associated structures. The application of AI in OMFS offers promising opportunities for improved surgical planning, precision, and patient outcomes. By leveraging advanced algorithms and machine learning techniques, AI systems can analyze complex medical imaging data, such as computed tomography (CT) scans and three-dimensional (3D) models, to aid in preoperative assessments, virtual surgical simulations, and the creation of personalized treatment plans. Furthermore, AI can assist surgeons in intraoperative decision-making by providing real-time guidance and feedback, enhancing surgical accuracy, and reducing complications [7,8].

In this review article, we delve into the diverse applications of AI in OMFS, exploring the potential of this emerging field to transform the way surgical procedures are performed and patient care is delivered. By analyzing recent advancements, challenges, and future prospects, we aim to provide a comprehensive overview of the current state of AI in this specialized domain of healthcare.

## 2. Materials and Methods

The authors a thorough literature review on the PubMed/MEDLINE and Embase databases, covering articles published in the English language from the inception of the databases until 1 December 2022. The search terms used were (1) “OMFS” OR “oral and maxillofacial” OR “oral and maxillofacial surgery” OR “oral surgery” AND (2) “AI” OR “artificial intelligence”. The search format was customized to each database’s ideal syntax. Furthermore, a thorough examination of the reference lists of each retrieved article and systematic review was conducted in order to identify relevant information.

## 3. Artificial Intelligence in Medicine

AI characterizes computer systems that can replicate and imitate human reasoning and thinking, learn from acquired knowledge, and make judgments. Machine learning, a subset of AI, involves using mathematical models to enable computers to learn without explicit programming [9]. This process utilizes structured and semi-structured data to train machine learning models, enabling them to generate accurate results or predictions. As the computer system gains experience, it continues to learn and improve over time.

This comprehensive review delineates the multifarious AI applications within the realm of OMFS, unfurling the latent potential of this burgeoning domain in reshaping surgical procedures and patient-centric healthcare delivery. By dissecting contemporary advancements, surmountable hurdles, and future trajectories, our objective resides in presenting a holistic overview of the extant state of AI in this specialized echelon of healthcare.

**Figure 1:**
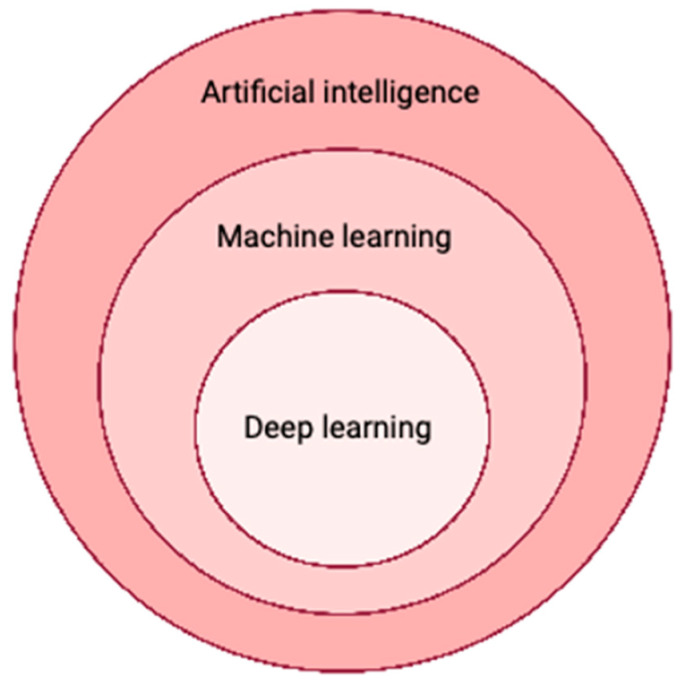
Artificial intelligence leverages different concepts such as machine learning and deep learning.

Deep learning, characterized by neural network models with multiple layers of interconnected nodes that can predict outcomes, represents one of the most complex forms of machine learning. Owing to the faster processing capabilities of modern graphics processing units and cloud architectures, deep learning can uncover thousands of hidden features within these models. In the healthcare industry, deep learning is widely employed for the identification of potentially malignant tumors in radiology images [10]. Particularly in the field of radiomics, which focuses on detecting clinically relevant features in imaging data beyond the capabilities of the human eye, deep learning has become a promising tool for oncology-oriented image analysis [11]. The combination of deep learning algorithms with radiomics holds great promise for improving diagnostic accuracy, surpassing the performance of previous generations of automated image analysis tools such as computer-aided detection systems (Figure 1).

## 4. Artificial Intelligence for Clinical Scenarios 

AI has made significant advancements in the field of medical sciences, encompassing distinct applications that revolutionize healthcare. These applications include matching patient symptoms with the appropriate physician, medical diagnosis, medical prognosis, drug discovery, automated language translation, and organizing medical records, notes, and images [12,13,14,15,16].

One notable area of progress is represented by the application of AI systems in diagnostics. In disciplines such as dermatology, AI utilizes clinical imaging data to develop classification models that assist physicians in diagnosing conditions like skin cancer and lesions as well as psoriasis [17,18]. For instance, Esteva et al. trained a deep convolutional neural network (DCNN) model using 129,450 images to classify malignant melanoma or benign nevus, as well as keratinocyte carcinoma or seborrheic keratosis [13]. Interestingly, the DCNN model performed with 71% accuracy, at the same level as 21 board-certified dermatologists (66% accuracy), showcasing its proficiency in accurately classifying skin malignancies. Not only did these AI systems demonstrate comparable diagnostic abilities to dermatologists, but they also required significantly less time for training. 

Beyond diagnostics, AI technologies have been used for enhancing patient care and improving the patient experience. AI assistants, such as those developed by businesses like BotMD, provide around-the-clock clinical support, bridging the gap between patients and healthcare providers. These systems offer valuable assistance in clinical decision-making and facilitate access to clinicians across multiple institutions, ensuring prompt and efficient medical care.

## 5. Artificial Intelligence in Oral and Maxillofacial Surgery

The application of AI in OMFS has gained significant popularity in recent decades, contributing to various aspects of the specialty. AI has been utilized for tasks such as diagnosis, cephalometrics, preoperative planning, intraoperative measurements, outcome evaluation, and postoperative follow-up in OMFS [8,19].

One area where AI algorithms have shown promise is in optimizing the management of impacted teeth. For instance, an AI-based model can aid in determining the surgical indication for tooth extraction by predicting the likelihood of tooth eruption-related risks [20]. Moreover, AI-aided tools have been deployed to assess the surgical difficulty of planned procedures preoperatively [21]. AI algorithms trained with clinical input data could predict the success of osteointegration procedures and dental implants, as well as optimize the design of dental implants prior to surgery [22].

**Figure 2:**
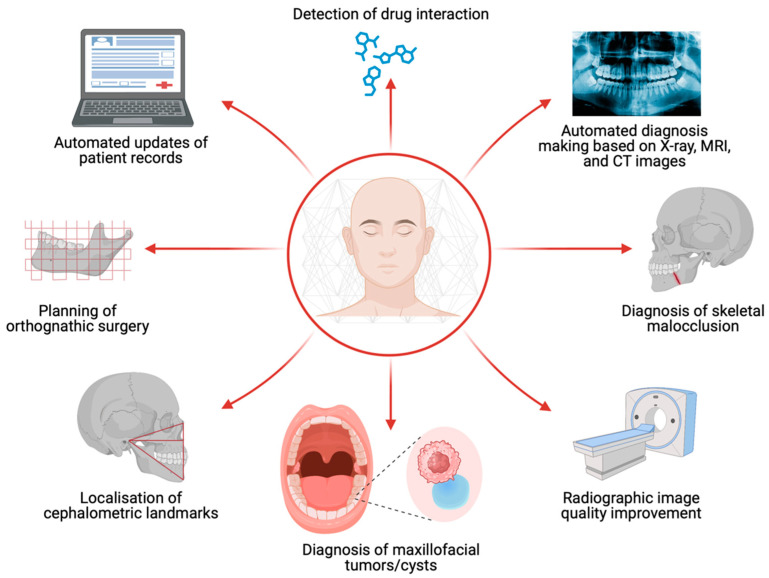
Artificial intelligence (AI) can be used for a wide array of clinical scenarios in oral and maxillofacial surgery. For instance, AI can facilitate the diagnosis of maxillofacial tumorous lesions and enhance the localization precision of cephalometric landmarks.

AI has also found application in robotic surgery, within the field of OMFS. AI-aided cranial surgical procedures, including dental implants, tumor resection, biopsies, and temporomandibular joint surgery, have demonstrated successful outcomes [8,23,24,25]. Research has shown that AI-aided surgery improved the accuracy and safety of oral implant procedures compared to traditional freehand techniques. Specifically, the use of AI reduced the need for revision surgeries and implant repositioning in some cases [26]. Furthermore, AI-aided approaches enabled more precise surgical resection of tumors and cysts, minimizing the requirement for additional procedures [27]. Distinct AI applications within the field of OMFS are depicted in Figure 2. Table 1 demonstrates the unique modalities that AI can be utilized through to aid in OMFS.

### 5.1. Radiographic Image Quality Improvement 

The quality of radiographic images can often be compromised by distinct interferences during the image acquisition and transmission process [28,29]. Deep learning techniques have shown great potential in overcoming the limitations of conventional methods, such as sparse-based or filtering techniques, for image quality improvement. A valuable application of deep learning lies in image denoising for enhancing the quality of low dose CT images. Convolutional neural network and generative adversarial network architectures have been utilized specifically for noise reduction and image deblocking tasks [30,31]. The integration of deep learning into low-dose CT research is a recent development aimed at minimizing patient exposure while maintaining image quality. Experiments are currently underway to leverage deep learning and machine learning technologies for denoising purposes, with the goal of maximizing the potential benefits in low-dose CT applications [32].

Another significant concern in diagnostic imaging is the presence of motion artifacts caused by patient or organ movements [33]. AI has been employed in dental CT and cone beam CT (CBCT) images to reduce metal artifacts. Dental crowns and implants, which have high attenuation coefficients, can cause scattering, photon starvation, and beam hardening phenomena, resulting in the formation of dark and bright streak artifacts [34]. These artifacts can negatively impact the accuracy of diagnostic procedures. By leveraging AI techniques, such as advanced image reconstruction algorithms and deep learning approaches, the presence of metal artifacts in dental CT and CBCT images can be mitigated, leading to precision improvement of diagnostic outcomes [35].

### 5.2. Diagnosis of Maxillofacial Cysts and Tumors

Accurate classification and diagnosis of various maxillofacial cysts and tumors pose challenges for physicians. The integration of AI into the automated diagnosis of these conditions holds significant potential in clinical practice. Of note, there is already a variety of commercial programs (e.g., dentalXr (www.dentalXr.ai, accessed on 24 January 2023, dentalXrai GmbH, Berlin, Germany), or Dentomo (www.Dentomo.com, accessed on 24 January 2023, dezzai, Madrid, Spain)) available for diagnosing maxillofacial cysts, cavities, dental fillings, or root canal fillings. For instance, Abdolali et al. proposed a model that uses asymmetry analysis to automatically segment radicular cysts, dentigerous cysts, and keratocysts [36]. Similarly, Rana et al. employed a surgical navigation program to segment keratocysts and measure their volume [37]. Other researchers are focusing on developing AI models trained with 2D/3D images to classify maxillofacial lesions and tumors [38,39]. However, it is important to note that the initial phase of lesion detection still requires manual input, and developing a fully automated model capable of identifying cysts and tumors remains a challenge. Of note, in a systematic review including 13 studies, Santer et al. found that AI carries promising potential in detecting suspicious lymph nodes in patients with locally-advances head and neck squamous cell carcinoma. The authors showed that AI demonstrated a mean accuracy of 86% for lymph node detection [40].

### 5.3. Localisation of Cephalometric Landmarks and Diagnosis of Skeletal Malocclusion

The precise localization of cephalometric landmarks is critical for accurate orthodontic diagnosis and treatment procedures. Inconsistencies in landmark identification can negatively impact diagnoses and treatment outcomes. To address this issue, automated landmark detection using machine learning techniques on lateral cephalometric radiographs has emerged as a promising approach [41,42]. Previous studies have explored different methods for landmark detection in cephalometric radiographs. For example, Lindner et al. attempted to classify skeletal abnormalities by projecting landmarks in lateral cephalograms [43]. Wang et al. utilized multiscale decision tree regression voting with scale-invariant patch features for landmark detection, enabling the calculation of specific clinical parameters [44,45]. Additionally, some approaches have aimed to locate cephalometric landmarks without relying on machine learning. Grau et al. employed a line detection module followed by pattern-matching techniques for landmark detection [42]. In the Automatic Cephalometric X-Ray Landmark Detection Challenge, hosted at the Institute of Electrical and Electronics Engineers (IEEE), two machine learning methods demonstrated superior performance across various precision ranges [46]. Ibragimov et al. utilized game theory and random forests for landmark detection, achieving higher results within a narrower precision range [47].

Current techniques for fully automatic identification of cephalometric landmarks have yielded significant efficiency gains and increased the potential for widespread application [48]. Deep learning has garnered recent attention in this field. Previous research has shown that systems employing the random forest technique can immediately identify 19 landmarks. When adopting automatic cephalometric landmark detection in clinical practice, computational performance becomes crucial, especially when multiple landmarks need to be identified simultaneously [41]. Recently, there have been many new studies focusing on AI programs and their accuracy for cephalometric landmarks; however, there has not yet been a comprehensive systematic review or meta-analysis for this developing field.

The diagnosis and treatment planning for patients with dentofacial abnormalities often requires a combination of orthodontic and surgical interventions [49]. Skeletal analysis plays a crucial role in this process, utilizing posteroanterior and lateral cephalograms to evaluate the anteroposterior, lateral, and vertical dimensions of the jaws and face. In addition, accurate diagnosis heavily relies on the proper identification of landmarks on a cephalogram, which is essential for successful treatment outcomes [50]. However, traditional cephalometric landmark detection techniques have limitations, including time-consuming procedures, potential bias, and inaccuracies associated with detected landmark data, which can be influenced by the observer’s expertise [51,52].

In recent research, deep learning algorithms have been investigated for cephalometric analysis, showcasing promising methods for improvement. Moreover, AI has been applied to automatically detect and categorize skeletal malocclusions using 3D craniofacial scans. One possible way to help orthodontists choose the optimum course of patient treatment was proposed by Kim et al. in 2020. The method was developed using a convolutional neural network (CNN)-based deep learning method for bone categorization [53]. These AI-based networks have demonstrated over 90% sensitivity, specificity, and accuracy in vertical and sagittal skeletal diagnosis. Additionally, machine learning techniques have shown potential in identifying cephalometric predictors for the need for orthognathic surgery in patients with treated unilateral cleft lip and palate [54]. Therefore, the integration of AI into these processes may reduce the labor-intensive nature of manual assessment and increase diagnostic precision [55].

### 5.4. Planning of Orthognathic Surgery 

Precise preoperative planning is crucial for successful outcomes in orthognathic surgery (OGS). Of note, current standard programs for planning orthognathic surgery (e.g., IPS CasePlanner (KLS Martin, Tuttlingen, Germany)) are commonly based on non-AI concepts. Traditional 2D surgical planning methods utilizing radiographs and models have limitations, particularly for patients with extensive facial asymmetry. These methods can lead to hazards such as bone contact issues and inconsistent pitch roll and yaw rotation in 2D designs [56,57]. However, advancements in 3D imaging have revolutionized the field by introducing computer-aided surgical simulation using CBCT images. 

Computer-aided planning based on 3D imaging enables the simplification of cephalometric analysis, splint production, and operation simulation. It provides a clearer visualization of dental abnormalities like yaw rotations, occlusal plane canting, and differential length of the body/ramus of the mandible [58]. The integration of virtual surgical planning, enabled by 3D imaging and 3D printing, provides surgeons with enhanced visualization of anatomical structures and has led to substantial improvements in treatment outcomes [59,60]. There is significant room for AI growth for planning orthognathic surgery and complementing current methods of 3D imaging and 3D printing.

### 5.5. Additional Benefits

AI has also shown promise in improving efficiency amongst surgeons and correcting potential errors within the context of OMFS. For example, it has been shown to play a role in automated diagnosis-making based on radiology, and automated updates of patient records. This may improve efficiency for surgeons; however, there may be accuracy issues for automated diagnosis based on radiology that still need further research and refinement. Finally, AI may also improve patient safety by detecting drug interactions, and correct potential prescribing errors.

## 6. Discussion

AI has rapidly evolved medical technology, and has made a significant impact within the field of OMFS. This review synthesized the latest literature regarding AI in OMFS and found eight distinct AI applications within the field of OMFS. These include radiographic image quality improvement, diagnosis of maxillofacial tumors/cysts, localization of cephalometric landmarks, planning of orthognathic surgery, diagnosis of skeletal malocclusion, automated diagnosis making based on radiographic findings, automated updates of patient records, and detection of drug interactions. In addition, this review presents practical implications of AI for use in clinical practice, as well as limitations of AI to be wary about.

One limitation of AI systems is their lack of transparency in explaining their decisions. As these models become more effective, they also become more difficult to understand. Transparency is essential in healthcare. The implementation of accurate yet enigmatic diagnostic and prognostic algorithms may be met with skepticism from patients and healthcare professionals. To promote adoption and transparency in treatment, the development of explainable AI models and the involvement of competent clinicians are necessary.

Most AI models rely on retrospective data for training and algorithm development. If these models are presented with new data that differ from the training dataset, their performance may be compromised. Additionally, a significant proportion of AI studies are not published in reputable peer-reviewed journals. It is crucial to evaluate AI systems on reliable, prospective databases and publish findings in peer-reviewed journals to validate their capabilities and facilitate their implementation in healthcare settings. Relevant information regarding positive and negative predictive values, as they relate to variations in patient outcomes, should be included in AI studies. OMFS surgeons should acquire a working knowledge of essential AI endpoints and metrics, focusing on practical application and patient outcomes rather than theoretical model accuracy in medical care.

It is recommended that OMFS residency programs incorporate essential AI curricula into their educational offerings to enhance understanding of AI models and algorithms. Program directors can invite healthcare AI experts to give lectures at a fundamental, clinical level. By doing so, residents can safely integrate AI technologies into their practice and critically evaluate models for their application. OMFS residents should be encouraged to contribute high-quality, prospective research during their training to improve future AI models. By basing AI models on more predictable future databases, the field can smoothly transition to AI use when it becomes a standard treatment. Collaboration among OMFS surgeons on an international level can generate high-quality AI training data from diverse patient populations, ensuring their continued important role in the utilization and innovation of AI in the maxillofacial region.

AI has countless potential applications in OMFS. By incorporating AI into diagnostic procedures, diseases, abnormalities, and fractures can be recognized more easily. High-quality database models can provide significant prognostic indicators that aid in surgical planning and aftercare. Implantology, for instance, could be revolutionized by creating a model that calculates the likelihood of implant failure by analyzing imaging data and considering medical comorbidities, medications, and other potential risk factors. 

The U.S. Food and Drug Administration has begun establishing a framework to ensure the safety and effectiveness of AI models in advancing patient care [61]. Due to the inherent learning nature of AI systems, they may continuously evolve and improve, necessitating a thorough review process. Criteria for performance monitoring should be established to calibrate AI models effectively.

OMFS surgeons can underline their leading role in the field of dental implanting by creating fast and precise AI-aided clinical models. With access to a wide range of dental imaging procedures, OMFS can play a larger role in a patient’s overall medical treatment (e.g., using AI to screen for various pathologies in panoramic imaging of the head and neck area). By understanding the limitations of, and staying informed about, regulatory developments, OMFS surgeons can be at the forefront of utilizing AI in OMFS diagnosis and prognosis. Integrating AI education and training into residency and other teaching programs, OMFS surgeons can contribute to advancing the field into a new era of technology.

Limitations of AI within the field of OMFS, and within medicine in general, are heavily debated. Ethical considerations as well as data privacy concerns are two of the foremost issues when it comes to integrating AI into medicine. AI, being a form of technology that is able to adapt on its own, provides the benefit of updating itself to better support medicine; however, it may also lead to model drift and start to program itself in a way that negatively impacts patient care. Furthermore, the Black Box problem, where people are unable to see how AI makes its decisions, further contributes to the distrust that people may have towards AI currently.

Future directions include further refining AI technology to further improve efficiency and accuracy within OMFS. This can include prospective trials implementing AI into workflows and quantifying its impact on patient care as well as identifying areas of improvement for AI. In this field, Wilke et al. are investigating imaging and treatment strategies for individuals diagnosed with head and neck cancer. To this end, the researchers are utilizing the HyperSight CBCT imaging technology and adaptive planning software that is backed by artificial intelligence. The study evaluates the image quality and artifact reduction of HyperSight CBCT in comparison to conventional CBCT and preoperative CT. Further, this study seeks to improve accuracy and effectiveness in radiation therapy by incorporating daily imaging. A group of researchers from the National Taiwan University Hospital is currently working on an automated diagnostic system for blepharoptosis that utilizes a machine learning-based framework to support telemedicine. This system aims to facilitate early screening of congenital/childhood ptosis, thereby enabling timely referral and treatment. 

**Figure 3:**
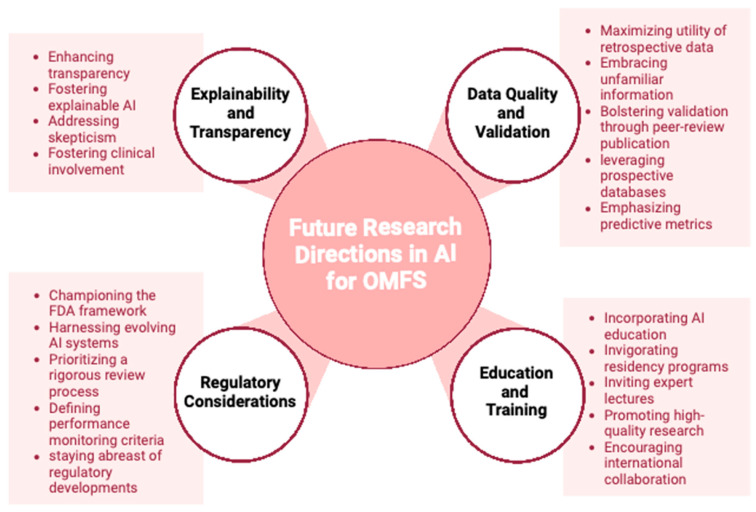
The fast-evolving topic of artificial intelligence (AI) in oral and maxillofacial surgery carries promising potential for future research projects such as establishing regulatory guidelines and strengthening the transparency of the AI algorithm.

A mounting body of evidence has underlined the clinical potential of AI technologies for different surgical and non-surgical specialties. The symbiosis of data-processing capacities, automatized learning strategies, and computational versatility represent a novel pathway towards cost and resource efficiency and improved patient care in OMFS. With the recent advent of chatbots, the next generation of AI tools is on the verge of further advancing OMFS care. While different AI strategies have already been implemented into the clinical management of OMFS and future studies are underway, it is pivotal to expand the interlinking between OMFS and AI technology and dismantle bureaucratic hurdles. In this context, clear legal frameworks, AI-qualified personnel, and state-of-the-art IT interfaces remain persisting challenges that slow down the implementation of AI in OMFS. Overall, AI carries untapped potential to strengthen the field of OMFS on multiple levels (Figure 3). This line of research may serve as an up-to-date primer for AI-interested OMFS surgeons and facilitate the use of AI technologies for improving surgical outcomes and patient care in OMFS.

## Figures and Tables

**Table 1:** Unique Modalities of AI Utilization.

AI Use	Description
Diagnosis and Treatment in OMFS	AI aids in diagnosis, cephalometrics, preoperative planning, intraoperative measurements, outcome evaluation, and postoperative follow-up. Also assists in managing impacted teeth, predicting tooth eruption risks, assessing surgical difficulty, predicting osteointegration success, and designing dental implants.
Robotic Surgery in OMFS	AI assists in cranial surgical procedures such as dental implants, tumor resection, biopsies, and temporomandibular joint surgery. AI enhances accuracy, reduces need for revisions, and ensures precise surgical resections.
Radiographic Image Enhancement	AI and deep learning improve radiographic image quality by reducing noise and deblocking, especially in low-dose CT images. Also, AI reduces motion artifacts and metal artifacts in dental CT and CBCT images.
Diagnostic AI Programs	Commercial programs such as dentalXr and Dentomo aid in diagnosing maxillofacial issues. AI is utilized to automatically segment and diagnose various cysts and tumors, and measure their volume.
Cephalometric Landmark Detection	AI and machine learning techniques automate landmark detection in cephalometric radiographs. Deep learning assists in this field, and AI algorithms help in detecting skeletal malocclusions using 3D craniofacial scans.
Surgical Planning	While current standard programs are non-AI, advancements in 3D imaging have enabled better surgical simulation. Computer-aided planning simplifies the surgical planning process and provides enhanced visualization. There is potential for AI growth in this area.
Efficiency and Safety in OMFS	AI can improve efficiency among surgeons by aiding in automated diagnosis based on radiology and updating patient records. It can also enhance patient safety by detecting drug interactions and correcting potential prescribing errors.
AI-powered apps	Promote optimal therapeutic compliance, ensuring adherence to treatment plans.
Smartphones with embedded biosensors	Continuous monitoring of physiological metrics for health prognostication.
Advanced AI algorithms	Analyze complex medical imaging data like CT scans and 3D models for preoperative assessments, surgical simulations, and personalized treatment plans.
Deep Learning Models	Identification of tumors, especially in oncology-oriented image analysis.
AI-assisted diagnosis-making	Match patient symptoms with appropriate physicians, medical diagnosis, prognosis, drug discovery, language translation, and organizing medical records.
Deep Convolutional Neural Network (DCNN) model	Diagnosis of skin conditions such as malignant melanoma, benign nevus, keratinocyte carcinoma, and seborrheic keratosis.
AI Assistants (e.g., BotMD)	Provide around-the-clock clinical support, bridging the gap between patients and healthcare providers, assisting in clinical decision-making.

## Data Availability

Not applicable.
